# What do women with epilepsy know about pregnancy?

**DOI:** 10.4102/safp.v66i1.5937

**Published:** 2024-08-16

**Authors:** Miyalani G. Baloyi, Rethabile Khalema, Sumaiya Adam

**Affiliations:** 1 Department of Obstetrics and Gynaecology, Faculty of Health Sciences, University of Pretoria, Pretoria, South Africa

**Keywords:** epilepsy, anti-epileptic drugs, knowledge, pregnancy, women of reproductive age, preconception counselling

## Abstract

**Background:**

Understanding the intersection of epilepsy and pregnancy, including knowledge gaps and healthcare access for women with epilepsy (WWE), is critical. This study evaluated WWE knowledge gaps and information needs concerning epilepsy’s impact on their sexual and reproductive health during pregnancy and examined healthcare system factors affecting their access to information, aiming to identify areas for improvement in educational and healthcare strategies to enhance health management for WWE.

**Methods:**

From July 2022 to June 2023, 111 WWE aged 18 to 40 years were recruited from the family medicine and internal medicine outpatient departments at Steve Biko Academic Hospital, Tembisa Tertiary Hospital (TTH), and Kalafong Hospital. Interviews assessed various aspects related to epilepsy in pregnancy and contraceptive use.

**Results:**

The study found strong links between WWE, their demographics, and their awareness of pregnancy-related epilepsy issues. Participants from TTH showed notably higher awareness (85.5%) of risks from epilepsy and AED during pregnancy (*p* < 0.05). Age and education significantly influenced pregnancy planning and understanding of medication risks. Younger women (20–25 years) were more inclined towards future pregnancies, and those with more education were better informed about medication risks (*p* < 0.05); and 68.5% had received counselling on AED and contraceptive interactions, yet only 16.2% knew AED could reduce contraceptive effectiveness.

**Conclusion:**

The study reveals significant knowledge gaps in WWE regarding the impact of epilepsy and AED on pregnancy, suggesting tailored educational and counselling initiatives to improve WWE health outcomes and quality of life, advancing chronic disease management and public health objectives.

**Contribution:**

The study highlights substantial knowledge gaps in epilepsy during pregnancy among WWE, urging tailored counselling and information to empower informed decisions.

## Introduction

The International League Against Epilepsy (ILAE) task force defined epilepsy as a disorder of the brain characterised by any of the following: (1) at least two unprovoked or reflex seizures more than 24 h apart; (2) one unprovoked or reflex seizure with a recurrence risk of at least 60% over the next 10 years, similar to the risk after two unprovoked seizures; (3) diagnosis of an epilepsy syndrome.^1^ The World Health Organization (WHO) estimates that epilepsy contributes just under 1% to the global disease burden.^2^ There is a disproportionate geographical burden of epilepsy, with 80% of cases living in low- or middle-income countries (LMICs), and 20% of the global burden is in Africa.^3^ In sub-Saharan Africa, persons with epilepsy face multiple challenges, including stigmatisation and associated cultural barriers, scarcity of diagnostic capacity, and inadequate supply of anti-epileptic drugs (AED), resulting in a more than 70% treatment gap.^3,4^ Diagnosis of epilepsy and the use of AED present women of childbearing age with unique challenges, fear, misunderstanding, discrimination, and social stigma, which negatively impact the quality of their lives and that of their families.^5^

The prevalence of epilepsy appears higher in sub-Saharan Africa compared to other regions, although estimates vary significantly for unclear reasons.^4^ However, we do know that approximately 40% of women with epilepsy (WWE) are of reproductive age, that is between 19 years and 44 years of age.^4^ The challenges faced by WWE in their reproductive age include the effects of the seizures and AED on hormonal function, potentially affecting their sexual and reproductive health.^6^

It has been found that WWE who attend antenatal clinics often lack sufficient information about their condition. Typically, they first receive advice during these visits, which is too late for making informed decisions about critical matters such as contraceptive options, timing of pregnancy, pre-pregnancy supplementation, and review of AED.^6^ Many WWE can conceive and have healthy children.^4,6^ Certain AED are linked to pregnancy complications, including changes in drug clearance, reduced cognitive function in the foetus, and a higher risk of major congenital malformations.^6^ Effective contraception is crucial for WWE of childbearing age because of the teratogenic risks and hormonal interactions associated with AED.^7,8,9^

In addition to the challenges mentioned earlier in the text, previous studies reveal a lack of knowledge among WWE and the healthcare professionals involved in their care.^6,7^ Women with epilepsy who are of reproductive age have differing information and treatment needs compared with men of the same age group.^6^ Knowledge regarding appropriate contraception, the use of folic acid prior to and during pregnancy, and the ideal seizure-free period prior to conception is of paramount importance.^8,9^ Knowledge of the AED which they take, the effects of AED on the pregnancy, and the effects of pregnancy on the disease are essential.^8^

Given the challenges faced by WWE, knowledge of their disease is an important factor in optimising the control of their seizures. Better-informed patients participate more easily in the treatment process, thereby reducing disease-related anxiety and coping better with stigma.^1,10^ Many studies have demonstrated that education and counselling can increase AED compliance, lead to better AED tolerance, and result in fewer side effects.^7,10,11^ In addition, the counselling offered in the preconception period can help increase knowledge of WWE to make informed decisions regarding pregnancy and reduce the incidence of foetal abnormalities.^11,12^

This study aimed to assess the knowledge and understanding among WWE about how the condition affects their sexual and reproductive health, particularly during pregnancy. It sought to identify their needs for information, areas where they lack knowledge, and factors within the healthcare system that impact their access to information. The goal was to highlight these elements to improve educational and healthcare strategies for WWE.

## Methods

### Study design

Between July 2022 and June 2023, a cross-sectional analytical study was conducted at outpatient clinics in internal medicine and family medicine at three hospitals in Steve Biko Academic Hospital, Tembisa Tertiary Hospital, and Kalafong Hospital. Steve Biko Academic Hospital in Pretoria, Tembisa Tertiary Hospital in Ekurhuleni, and Kalafong Hospital in Atteridgeville are major tertiary hospitals in Gauteng that provide specialised care, and serve diverse, often underserved populations, Over the study period, each clinic consulted approximately 60–100 WWE.

### Study population

A total of 111 WWE were invited to participate in the study based on consecutive sampling. Consecutive WWE between the ages of 18 years and 40 years on AED with a diagnosis of epilepsy for at least 6 months were invited to participate. Women with epilepsy with cognitive impairment, as determined by their caring physician, were excluded from the study because of challenges of obtaining informed consent and correctly assessing their knowledge. The WWE younger than 18 years were excluded because of challenges in obtaining parental consent.

### Sample size

To achieve an estimation accuracy of within 10% for proportions, we established a minimum sample size of 97 participants. This size was calculated using nQuery version 8.9 (Boston, Massachusetts, United States [US]), based on the conservative assumption that expected proportions would be 50%. The study assessed the relationship between outcomes and exposures through multivariable logistic regression. The sample size was increased to 111 participants to adequately evaluate three exposure variables and up to five demographic and/or clinical variables, ensuring that the number of events per variable (EPV) exceeded 5.

The binary outcomes of Objective 1 (epilepsy history) were estimated with a precision of 10% accuracy from at least 97 participants. The relationships of these outcomes with the exposures in Objective 2 (fertility wishes, contraception usage, knowledge of effect of epilepsy on pregnancy, knowledge of effect of pregnancy on epilepsy) were analysed using multivariable logistic regression. This analysis confirmed that the sample size was sufficient for including three exposure variables and no more than five demographic/clinical variables, provided that the EPV was greater than 5.

### Measurements

Patients, who met the inclusion criteria, were approached by the researcher to participate in the study while awaiting their consultations for their routine follow-up appointment at their respective clinics. The interview was conducted in a private cubicle. If there was any question about their cognitive ability to participate, the caring physician was consulted and the interview was conducted after the consultation, if appropriate. The principal investigator administered a structured questionnaire in English, Setswana, or IsiZulu. For patients who spoke other languages, a medically trained colleague, who was briefed on the study and the questionnaire, and who was fluent in those languages served as a translator without receiving compensation. Interviews were 15 min – 20 min long, conducted in a private area at the clinics, and while not recorded, responses were documented in real time. Participation was voluntary, contingent on informed consent, and uncompensated.

Because of the lack of validated questionnaires suitable for this research, a self-developed tool was created based on the existing literature. This questionnaire included both open- and closed-ended questions. The first section gathered demographic data such as age, parity, race, marital status, education level, and epilepsy history. The second section focused on the participants’ epilepsy histories, including diagnosis, seizure frequency, and treatment history, while the third section assessed their understanding of the effects of pregnancy on epilepsy and epilepsy on pregnancy, using open-ended questions to avoid bias.

The open-ended questions covered topics such as: (1) interactions between AED and oral contraceptives, (2) the impact of pregnancy on AED effectiveness, (3) effects of AED on the foetus, (4) knowledge about pregnancy-related vitamin supplementation, (5) ideal seizure-free interval before pregnancy, (6) risk of foetal anomalies because of AED, and (7) likelihood of complications during pregnancy for WWE compared to those without. The data from open-ended questions were categorised for quantitative analysis.

#### Pilot study

The questionnaire used in the study was self-developed, and to ensure its quality, a pilot study was conducted before the main study commenced. The pilot aimed to test the feasibility of the study and refine the questionnaire. It involved a small sample size – 10% of the projected total of 111 participants, which equated to 11 patients – to assess the data-collection tools and methods for appropriateness, accuracy, validity, and reliability. This preliminary phase allowed for adjustments to the tool based on feedback and the identification of any weaknesses. The revised questionnaire was then piloted across hospitals on 12 patients, with 4 WWE from each hospital. Ambiguous questions were improved through consultations with maternal-foetal medicine specialists. Data from these participants were excluded from the final analysis.

Following the interviews, patients received information regarding pregnancy and reproductive health.

### Statistical analysis

Data were analysed using IBM^®^ SPSS^®^ statistics (version 27). The study summarised predominantly discrete data using frequencies, proportions, percentages, and 95% confidence intervals (CI). It assessed relationships between outcome and exposure/demographic/clinical parameters using Pearson’s Chi-square test, presenting results as crude odds ratios (OR) with 95% CI. Multivariable logistic regression was used to adjust these ORs. All statistical tests were conducted at a significance level of 0.05. Descriptive and inferential statistics, including chi-square tests, were applied, with a hypothesis rejected at a *p*-value less than 0.05.

### Ethical considerations

Ethical clearance to conduct this study was obtained from the University of Pretoria, Faculty of Health Research Ethics Committee (No. 157/2022). Written informed consent was obtained prior to including WWE in the study. Confidentially was assured by not recording any patient identifiers (name, date of birth, contact details) on the data-collection tool. Data were stored on a secure, password-protected Google Drive, accessible only to the principal investigator and the supervisor.

## Results

A total of 121 WWE were recruited for the study; 111 (91.7%) met the inclusion criteria and consented to participate in the study. Thirty-nine WWE (35.1%) were from Hospital 1, 27 (24.3%) were from Hospital 2, and 45 (40.5%) were from Hospital 3. Sixty (54.1%) WWE were from the internal medicine clinic, and 51 (45.9%) from the family medicine clinic. The women were between the ages of 26 years and 40 years (mean 28.6), 60 (54.1%) WWE had completed secondary school and 76 (68.5%) WWE had no family history of epilepsy. Most family members with epilepsy were mothers (*n* = 11; 9.9%) and brothers (*n* = 11; 9.9%). Only 38 WWE (34.2%) had not experienced seizures in the last 6-months (Table 1).

**TABLE 1:** Demographic characteristics of women with epilepsy (*N* = 111).

Variable	Description	*n*	%
Age (years)	18–20	15	13.5
	20–25	23	20.7
	26–30	33	29.7
	31–35	19	17.1
	35–40	21	18.9
Parity Range 0–5 Median 1	0	31	27.9
	1	27	24.3
	2	26	23.4
	3	16	14.4
	4	9	8.1
	5	2	1.8
Education	Completed secondary school	60	54.1
	Did not complete secondary school	23	20.7
	Tertiary education	28	25.2
Employment status	Full-time	35	31.5
	Part-time	20	18.0
	Unemployed	48	43.2
	Student	8	7.2
Age at diagnosis of epilepsy (*n* = 110)	1–10 years	19	17.1
	10–20 years	71	64.6
	20–30 years	16	14.4
	> 30 years	5	3.9
When was your last seizure?	< 1 month	27	24.3
	1–6 months	46	41.3
	6–12 months	24	21.6
	> 1 year	14	12.8
Management of epilepsy	Sodium valproate only	17	15.3
	Levetiracetam only	19	17.1
	Lamotrigine only	20	18.0
	Carbamazepine	5	4.5
	Polytherapy	48	43.2
	Unsure of treatment	2	1.8

Note: Age (years) – range = 18–40, mean = 28.6; Parity – range = 0–5, median = 1.

### Diagnosis and management

A significant number of women in the study were diagnosed with epilepsy during their childhood or adolescence. Specifically, 19 (17.1%) were diagnosed before the age of 10 years and 71 (64%) were diagnosed between the ages of 10 years and 20 years. Of those diagnosed in their teenage years, 28 (39.4%) received their diagnosis following recurrent seizures. Idiopathic epilepsy was diagnosed in 87 (78.4%) WWE, while 4 (3.6%) WWE developed epilepsy secondary to underlying conditions such as meningitis, stroke, or trauma. Twenty (18%) expressed uncertainty regarding both the diagnostic process and the aetiology of their epilepsy.

Regarding the medication used for managing epilepsy, all the WWE in the study were on AED. Sixty-one (55%) of WWE were on monotherapy, while 48 (43.2%) were on a combination of AED, with lamotrigine/levetiracetam being the most common combination (*n* = 29, 26.1%). Three WWE were on three (2.7%) AED and two (1.8%) WWE did not know which AED they were being treated with. The majority of the WWE, 62 (55.9%), reported experiencing side effects from their medication. The most common side effect being headache (*n* = 25, 29%) followed by poor concentration (*n* = 20, 23%) and fatigue (*n* = 15, 17%).

### Contraceptive use

The majority of (*n* = 80, 72.1%) WWE were using contraceptives, while 31 (27.9%) were not utilising any form of contraception. The most commonly utilised contraceptive was the intrauterine contraceptive device (intrauterine contraceptive device [IUCD]: *n* = 38, 34.2%), followed by injectables (*n* = 26, 3.4%), and the contraceptive implant (Implanon: *n* = 8, 7.2%). Additionally, four (3.6%) WWE had undergone bilateral tubal ligation, and four (3.6%) WWE were using combined oral contraceptives (COC). Thirty WWE (27%) expressed a desire for future pregnancies, although they did not specify when they planned to conceive. Of these 30 WWE, 13 (43.3%) were not using any form of contraception.

### Pregnancy

Sixty-seven WWE (61.3%) were unaware of how long a WWE should be seizure-free before attempting conception. Among the 44 WWE (39.6%) who knew that a seizure-free period of at least 6-months is recommended, 25 (56.8%) considered 6–12 months adequate, and 19 (43.2%) believed that a 1-year seizure-free interval was necessary. In addition, 51 WWE (45.9%) identified an increased risk of seizures as the most common complication for WWE during pregnancy, while 6 WWE (5.4%) found that complications are more likely if epilepsy is uncontrolled.

Seventy-seven (69.4%) WWE were aware of the effects of epilepsy and AED on pregnancy, while 34 (30.6%) WWE did not know of any effects of epilepsy on pregnancy. Seventy-five (67.6%) WWE thought that having epilepsy and the use of AED in pregnancy increased their risk of giving birth to a child with an abnormality, especially neurological anomalies. Two (1.8%) WWE thought that their risk of miscarriage was increased. In addition to the increased risk of foetal abnormalities, 7 (6.3%) WWE believed that their seizure risk was increased in pregnancy and 2 (1.8%) WWE thought that the baby was at increased risk of epilepsy.

The majority of the WWE (*n* = 104; 93.7%) did not believe that all AED are equally likely to cause an abnormality in the baby. However, the results show that there is a strong association (*p* < 0.05) between women’s responses to this question and their level of education. Specifically, out of the 6 (5.4%) WWE who believed that all AED were equally likely to cause an abnormality in the baby, 4 (66.7%) of these women did not complete secondary school. This suggests that there may be a relationship between the level of education and understanding of the potential risks associated with AED during pregnancy. Additionally, 2 WWE (1.8%) indicated that AED should be discontinued upon confirming pregnancy, and 1 woman (0.9%) was unsure what should be done.

Seventy-seven WWE (69.4%) were aware that vitamins are necessary before and during pregnancy, whereas 34 WWE (30.6%) were not. Among those informed, the majority (*n* = 62; 80.5%) identified folic acid as the essential vitamin needed, while a smaller group (*n* = 15; 19.5%) did not specify folic acid. In addition, 24 (21.6%) WWE believed that both multivitamins and ferrous sulphate are specifically required during pregnancy for WWE.

When asked what they would do upon discovering their pregnancy, 109 WWE (98.2%) knew to book an appointment at the antenatal clinic as soon as possible and 40 WWE (36.7%) recognised that their pregnancies would be high-risk. One woman (0.9%) was unsure of what to do, while another (0.9%) indicated she would stop her AED.

### Counselling

A significant disparity exists between knowledge and counselling among the women surveyed. Out of the 111 WWE we interviewed, 71 (64%) reported having received counselling on pregnancy in the past, while 40 (36%) WWE denied any sort of counselling.

Despite 76 (68.5%) WWE receiving counselling about contraception and the interaction between AED and COC, only 25 (22.5%) WWE recognised this interaction. Meanwhile, 86 (77.5%) WWE were unaware that AED could reduce COC efficacy. A significant correlation (*p* < 0.05) was found linking awareness of AED effects on COC with parity, current contraceptive use, and receipt of information about COC.

**FIGURE 1:**
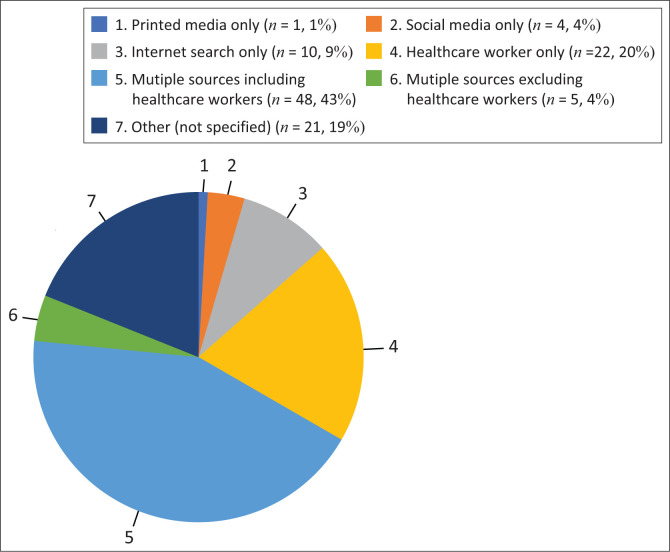
Where do women with epilepsy obtain information? (*N* = 111).

Figure 1 depicts the sources of information for WWE regarding their sexual and reproductive health. While most WWE (*n* = 53; 47.7%) relied on multiple sources, only 70 (63%) WWE obtained information from healthcare workers.

The survey results indicate that the preferred method of receiving information about pregnancy and epilepsy varies among the participants. The majority of the WWE (*n* = 62; 55.9%) favoured healthcare worker-led counselling sessions. Furthermore, 1 woman (0.9%) preferred a support group, 13 WWE (11.7%) opted for printed media such as information leaflets, and 5 WWE (4.5%) showed a preference for being directed to online resources. Moreover, 30 WWE (27%) expressed a desire for information through a combination of different sources. Notably, the same number of WWE (*n* = 30; 27%) specifically preferred to be counselled by a doctor.

### Factors influencing knowledge

In summary this study highlights significant correlations between demographic factors and knowledge about epilepsy-related pregnancy issues (Table 2):

- *Hospital influence*: Patients at Hospital 2 showed a higher awareness (85.5%) of the risks of continuing medication during pregnancy and the effects of epilepsy on pregnancy (77.8%), suggesting a strong influence of the treatment facility on patient knowledge with statistical significance.
- *Age-related differences*: There is a marked contrast in pregnancy planning between younger women (20–25 years, 73.9% planning pregnancies) and older women (31–35 years, 94.7% not planning pregnancies), with older WWE also demonstrating greater knowledge of prenatal vitamins. These differences are statistically significant.
- *Educational impact*: Higher education correlates with a better understanding of epilepsy’s impact on pregnancy, medication continuation, and prenatal vitamins, with 93.1% of post-school educated respondents showing awareness. The statistical significance of these findings underscores the role of education in health literacy.
- *Employment status*: Employment status impacts knowledge, with full-time employed and students more informed about pregnancy and epilepsy-related issues compared to unemployed individuals.
- *Family history*: A family history of epilepsy is associated with better knowledge about the effects of epilepsy and medications on pregnancy (82.4%), reflecting a significant impact of personal or familial experience.
- *Parity*: The number of children influences future pregnancy planning and knowledge, with those having more children less likely to plan more pregnancies and more informed about contraceptive interactions and prenatal vitamins. These associations are statistically significant.

**TABLE 2:** Factors affecting knowledge about epilepsy-related pregnancy issues (*N* = 111).

Variable	Side effects experienced			Future pregnancies planned			Current contraceptive usage			Counselled about pregnancy-related issues			Knows about the continuation of AED and its effects on pregnancy			Knows about the effects of epilepsy and AED on pregnancy			Knows about the interaction of AED and COC			Knowledge of supplements required in a pregnancy in WWE			Knowledge of complications during pregnancy in WWE			Knowledge of AED and foetal/neonatal affectation		
	*n*	%	*p*	*n*	%	*p*	*n*	%	*p*	*n*	%	*p*	*n*	%	*p*	*n*	%	*p*	*n*	%	*p*	*n*	%	*p*	*n*	%	*p*	*n*	%	*p*
**Hospital**	-	-	0.093	-	-	0.589	-	-	0.640	-	-	0.9	-	-	0.011*	-	-	0.05	-	-	0.175	-	-	0.342			0.305	-	-	0.861
1 (*n* = 39)	17	43.6	-	12	30.8	-	26	66.7	-	26	66.7	-	21	53.8	-	21	53.8	-	4	10.3	-	26	66.7	-	13	33.3	-	2	5.1	-
2 (*n* = 27)	19	70.4	-	12	44.4	-	20	74.1	-	19	70.4	-	23	85.2	-	21	77.8	-	7	25.9	-	22	81.5	-	14	51.9	-	2	7.4	-
3 (*n* = 45)	26	57.8	-	18	40.0	-	34	5.6	-	32	71.1	-	35	77.8	-	34	75.6	-	11	24.4	-	30	66.7	-	20	44.4	-	2	4.4	-
**Age**	-	-	0.656	-	-	< 0.001*	-	-	0.375	-	-	0.081	-	-	0.867	-	-	0.889	-	-	0.947	-	-	0.012*	-	-	0.814	-	-	0.424
18–20 (*n* = 15)	10	66.7	-	10	66.7	-	9	60.0	-	7	46.7	-	10	66.7	-	9	60.0	-	2	13.3	-	6	40.0	-	7	46.7	-	0	0.0	-
20–25 (*n* = 23)	14	60.9	-	17	73.9	-	16	69.6	-	13	56.5	-	16	69.6	-	17	73.9	-	5	21.7	-	15	65.2	-	10	43.5	-	0	0.0	-
26–30 (*n* = 33)	15	45.5	-	11	33.3	-	27	81.8	-	26	78.8	-	26	78.8	-	22	66.7	-	6	18.2	-	25	75.8	-	16	48.5	-	2	6.1	-
31–35 (*n* = 19)	11	57.9	-	1	5.3	-	15	78.9	-	14	73.7	-	13	68.4	-	14	73.7	-	4	21.1	-	18	94.7	-	7	36.8	-	2	10.5	-
35–40 (*n* = 21)	12	57.1	-	3	14.3	-	13	61.9	-	17	81.0	-	14	66.7	-	14	66.7	-	5	23.8	-	14	66.7	-	7	33.3	-	2	9.5	-
**Parity**	-	-	0.392	-	-	< 0.001*	-	-	< 0.001*	-	-	< 0.001*	-	-	0.552	-	-	0.699	-	-	0.020*	-	-	0.002*	-	-	0.008*	-	-	0.498
0 (*n* = 31)	20	64.5	-	20	64.5	-	10	32.3	-	11	35.5	-	20	64.5	-	19	61.3	-	1	3.2	-	13	41.9	-	6	19.4	-	0	-	-
1 (*n* = 27)	13	48.1	-	17	63.0	-	26	96.3	-	22	81.5	-	21	77.8	-	20	74.1	-	7	25.9	-	23	85.2	-	18	66.7	-	2	7.4	-
2 (*n* = 26)	13	50.0	-	5	19.2	-	23	88.5	-	23	88.5	-	20	76.9	-	20	76.9	-	5	19.2	-	22	84.6	-	12	46.2	-	1	3.8	-
3 (*n* = 16)	10	62.5	-	0	0.0	-	13	81.3	-	13	81.3	-	12	75.0	-	11	68.8	-	7	43.8	-	13	81.3	-	8	50.0	-	2	12.5	-
4 (*n* = 9)	6	66.7	-	0	0.0	-	6	66.7	-	6	66.7	-	6	66.7	-	5	55.6	-	1	11.1	-	6	66.7	-	3	33.3	-	1	11.1	-
5 (*n* = 2)	0	0.0	-	0	0.0	-	2	100.0	-	2	100.0	-	0	0.0	-	1	50.0	-	1	50.0	-	1	50.0	-	0	0.0	-	0	0.0	-
**Family history**	-	-	0.997	-	-	0.259	-	-	0.490	-	-	0.281	-	-	0.331	-	-	0.036*	-	-	0.369	-	-	0.162	-	-	0.801	-	-	0.290
No (*n* = 77)	43	55.8	-	26	33.8	-	57	74.0	-	51	66.2	-	53	68.8	-	48	62.3	-	17	22.1	-	51	66.2	-	32	41.6	-	3	3.9	-
Yes (*n* = 34)	19	55.9	-	16	47.1	-	23	67.6	-	26	76.5	-	26	76.5	-	28	82.4	-	5	14.7	-	27	79.4	-	15	44.1	-	3	8.8	-
**Education level**	-	-	0.686	-	-	0.448	-	-	0.124	-	-	0.013*	-	-	0.03*	-	-	0.013*	-	-	0.199	-	-	0.029*	-	-	0.005*	-	-	0.040*
Completed secondary school (*n* = 59)	34	57.6	-	25	42.4	-	42	71.2	-	37	62.7	-	39	66.1	-	39	66.1	-	12	20.3	-	40	67.8	-	24	40.7	-	1	1.7	-
Did not complete post-school (*n* = 23)	11	47.8	-	6	26.1	-	13	56.5	-	13	56.5	-	13	56.5	-	11	47.8	-	5	21.7	-	12	52.2	-	4	17.4	-	4	17.4	-
Tertiary (*n* = 29)	17	58.6	-	11	37.9	-	25	86.2	-	27	93.1	-	27	93.1	-	26	89.7	-	5	17.2	-	26	89.7	-	19	65.5	-	1	3.4	-
**Employment**	-	-	0.410	-	-	0.020*	-	-	0.062	-	-	< 0.001*	-	-	0.016*	-	-	0.013*	-	-	0.681	-	-	0.006*	-	-	0.026*	-	-	0.698
Full-time (*n* = 35)	22	62.9	-	10	28.6	-	31	88.6	-	32	91.4	-	32	91.4	-	30	85.7	-	8	22.9	-	31	88.6	-	21	60.0	-	2	5.7	-
Part-time (*n* = 20)	8	40.0	-	6	30.0	-	14	70.0	-	16	80.0	-	11	55.0	-	13	65.0	-	5	25.0	-	16	80.0	-	9	45.0	-	2	10.0	-
Unemployed (*n* = 48)	27	56.3	-	19	39.6	-	30	62.5	-	25	52.1	-	31	64.6	-	26	54.2	-	7	14.6	-	27	56.3	-	13	27.1	-	2	4.2	-
Student (*n* = 8)	5	62.5	-	7	87.5	-	5	62.5	-	4	50.0	-	5	62.5	-	7	87.0	-	2	25.0	-	4	50.0	-	4	50.0	-	0	0.0	-

AED, anti-epileptic drugs; COC, combined oral contraceptive; WWE, women with epilepsy.

* , Denotes statistical significance.

The regression analysis (Online Appendix 1) confirmed these observations, demonstrating that demographic factors such as age, educational level, and parity significantly affect planning for future pregnancies and knowledge about pregnancy and epilepsy. These findings are statistically robust, emphasising the need for tailored educational interventions based on these demographic characteristics to improve health literacy among WWE.

## Discussion

Women with epilepsy often have normal pregnancies and healthy deliveries, but they face specific challenges compared to women without epilepsy. These challenges necessitate detailed information about the heightened risks because of epilepsy and its treatments during childbearing years. Preconception guidance is essential, including advising on proper contraceptive use to postpone pregnancy until optimal health is achieved and ensuring consistent intake of prenatal supplements such as folic acid.^13,14^ A lack of knowledge about these practices can result in poorly informed reproductive choices, posing risks to both the mother and the child. An unplanned pregnancy is a missed opportunity to mitigate risks associated with teratogens and to optimise seizure management through careful selection of medications and reducing polytherapy, particularly with medications such as sodium valproate.^15,16^

This study, conducted on 111 WWE, provides comprehensive insights into their knowledge and awareness regarding pregnancy-related issues, the impact of epilepsy and AED, and their contraceptive practices. The analysis reveals significant correlations between various demographic characteristics and the level of knowledge these women possess, highlighting the need for improved education and preconception counselling to enhance their reproductive health outcomes.

The study highlighted a stark difference in pregnancy planning between younger and older women. Younger women appeared more likely to plan future pregnancies compared to their older counterparts, who, despite planning fewer pregnancies, exhibited greater knowledge about prenatal vitamins. This suggests a potential shift in focus from pregnancy planning to health management as women age.^17,18^

Having a family history of epilepsy significantly enhanced understanding of the disease’s implications during pregnancy. In addition, women with more children showed less inclination to plan more pregnancies and were more knowledgeable about contraceptive interactions and prenatal vitamins.

Education level was significantly associated with knowledge about epilepsy’s impact on pregnancy, medication continuation, and awareness of prenatal vitamins. Patients with higher education levels demonstrated better understanding and awareness, underscoring the importance of educational background in health literacy. Jasnos et al. found that one-third of interviewed WWE had insufficient knowledge about responsible motherhood, with this issue being most prevalent among women with the lowest level of education. Most women suffering from epilepsy become interested in issues related with motherhood only after they become pregnant whereas women with at least secondary education are more interested in issues of procreation and responsible motherhood.^19^

Employment status influenced the knowledge levels, with full-time employed and students showing better awareness compared to unemployed individuals. This might reflect broader access to resources and information because of better socioeconomic status.

This study showed that there was a significant association between WWE who had obtained information about pregnancy-related issues and taking contraceptives. Effective contraception is particularly important for WWE of childbearing age because of potential AED-related teratogenicity and hormonal interactions.^8,20^ In this study we illustrated a significant association between women’s knowledge of AED and COC interaction and their parity. It is recommended that WWE should be counselled regarding the choice of contraceptive and its interaction with AED much earlier in their reproductive years.^20,21^ In a German study conducted in 2018, 56% of women knew about the relationship between AED and COC.^22^ However, other studies have found that many women still do not receive the necessary information; a survey found that less than 7% of WWE received counselling on contraceptives and only 18% on pregnancy.^23,24,25^

Numerous studies indicate that high-dose folic acid is linked to neurodevelopmental advantages. Consequently, both the United Kingdom and South African maternity care guidelines recommend daily supplementation of at least 400 mcg of folic acid pre-conceptionally and throughout the first trimester of pregnancy.^8,9,26,27^ In contrast to our findings where only 55% of WWE expressed the importance of folic acid supplementation, research shows that WWE generally understand the importance of vitamin supplementation before and during pregnancy. This awareness is shaped by factors such as age, educational level, employment status, number of children, contraceptive use, and previously received information. However, the study also found that WWE, particularly those with lower educational backgrounds, often harbour misconceptions about the risks posed by seizure medications during pregnancy, mistakenly believing that all such medications might cause foetal abnormalities. This underscores the necessity of counselling and education as integral components of high-quality care, ensuring patients’ knowledge and comprehension of their condition and its effects are thoroughly assessed.^16^

The Ideal World survey reported that one-third of WWE who currently had children did not receive any information regarding the impact of their AED on their pregnancy and only 7% were referred for specialist care during this time.^28^ In our study, 64% of women were counselled specifically about pregnancy and 68.5% were counselled on the effect of AED on COC. Despite the counselling, 61.3% remained unaware of the ideal seizure-free interval for pregnancy planning and 26.1% were still on sodium valproate.

In our study, WWE from Hospital 2 demonstrated higher awareness about the risks associated with continuing AED during pregnancy and the effects of epilepsy on pregnancy. This finding indicates that the hospital at which patients were seen played a critical role in their knowledge about epilepsy-related pregnancy issues. Different hospitals may have varying protocols or effectiveness in educating patients about their conditions. The results highlight the need for targeted education and counselling for WWE, especially regarding the use of AED during pregnancy and their potential effects on both the mother and the foetus. This could help in making informed decisions about family planning, medication management, and pregnancy care. Morell et al. found that in cases where healthcare providers, who themselves were not sufficiently knowledgeable about the issues concerning reproduction and epilepsy, encountered WWE, inadequate counselling and sub-optimal care were more likely.^29^

While a causal relationship has not been established, inadequate patient knowledge is likely correlated with inadequate physician knowledge in this area.^16,20^ These endeavours should include education of the healthcare workers and creation of standardised content with evidence-based information. In their study, Li et al. found that the understanding of pregnancy-related knowledge did not differ between WWE and their caregivers, where knowledge in both groups was unsatisfactory.^30^ It is urgent for WWE and their caregivers to improve their pregnancy-related knowledge of epilepsy.^22^

In our study the majority of women preferred to receive information from a healthcare worker. Similarly, a study in Poland found that patients preferred to be counselled by their caring physicians.^19^ In contrast, Crawford and Hudson found that the majority (59%) of women preferred written information, 28% preferred information from a healthcare professional, and 4% via the Internet.^28^ This may reflect differing literacy levels and needs in high versus low-middle income countries.

Pallerla et al. found that women with poor knowledge about epilepsy and pregnancy have difficulty in reproductive decision-making, thereby increasing risk perception of childbearing.^6^ This, together with the findings of overall poor knowledge of WWE about the effect of epilepsy on contraception and pregnancy suggest a need for the healthcare system to proactively provide information and support to WWE. This includes improving access to preconception counselling and ensuring that WWE receive comprehensive and updated information about managing epilepsy during pregnancy. The study underscores the importance of healthcare workers being aware of the specific informational needs of WWE. Healthcare workers should engage in discussions about pregnancy planning and management with WWE and provide tailored advice based on the individual’s health status and treatment plan.

### Recommendations

The majority of information on this topic comes from high-income countries,^12^ and the findings of this study could guide future research directions aimed at developing effective educational interventions and policies to enhance knowledge and healthcare outcomes for WWE. Additional research is necessary to explore the barriers WWE face in accessing, receiving, and retaining appropriate information. This includes identifying the most effective methods for information dissemination and understanding the roles various healthcare providers play in this process.

To address these needs, several strategies could be implemented. Developing hospital-based educational programmes tailored to the demographic characteristics of WWE can ensure that all patients receive comprehensive counselling, regardless of their socioeconomic or educational status. Training healthcare providers across all facilities is crucial to deliver consistent information about the risks of epilepsy and AED during pregnancy, with an emphasis on the importance of continuing education and adherence to updated guidelines.

Community outreach initiatives might focus on engaging younger WWE, possibly in settings such as community colleges or workplaces, to enhance awareness about the importance of prenatal care and effective disease management. Increasing the availability of both digital and printed educational materials that cater to different literacy levels could also improve accessibility and understanding for all WWE.

Additionally, collaborating with educational institutions to integrate basic health education into curricula could promote awareness from a young age, focusing on chronic disease management, including epilepsy. These efforts could collectively contribute to better informed and prepared WWE, ultimately leading to improved health outcomes.

### Strengths and limitations

The strengths of this study are the utilisation of descriptive and inferential statistics to analyse the results, providing a quantitative approach to understanding the knowledge gaps among WWE. This allows for a more systematic and objective examination of the data, enhancing the rigor of the study. Additionally, the study involved a significant number of participants from three different hospitals and two different departments. This increases the diversity, generalisability, and representativeness of the findings.

Limitations in this study include the recruitment of patients from tertiary hospitals, as these patients typically have more severe or uncontrolled epilepsy and may not represent the broader population of WWE. To address these limitations, future studies could consider recruiting participants from a wider range of healthcare settings, including primary care clinics, to capture a more diverse population. Additionally, conducting the interviews solely by the principal investigator may have introduced bias, as this could have influenced participants’ responses, leading to potential bias. It would have been beneficial to have multiple interviewers to minimise this potential bias.

## Conclusion

This study has illuminated significant knowledge gaps among WWE regarding pregnancy, particularly in understanding the interactions between AED and contraceptives, and the broader impacts of epilepsy on pregnancy, emphasising the critical need for specialised education and counselling services tailored to their unique needs. A comprehensive, multidisciplinary approach involving healthcare providers, educators, and policymakers is imperative. Such an approach should aim to enhance educational programmes, support mechanisms, and research, all specifically designed to meet the distinct requirements of WWE during pregnancy. By strategically addressing these gaps through tailored educational interventions and consistent training for healthcare providers, we can significantly improve the health outcomes and quality of life for WWE. These efforts are not only crucial for the individuals directly affected but also align with broader public health objectives to mitigate the effects of chronic diseases through improved patient education and proactive self-management.
